# Time-Dependent Analysis of Blood Glucose Stability in Sodium Fluoride Versus Plain Blood Collection Tubes

**DOI:** 10.7759/cureus.119320

**Published:** 2026-11-08

**Authors:** Kuldeep K Gupta, Tabassum Yasmin, Farhat Khan, Priyanka Asia

**Affiliations:** 1 Biochemistry, Uma Nath Singh Autonomous State Medical College, Jaunpur, Jaunpur, IND; 2 Biochemistry, NKP Salve Institute of Medical Sciences and Research Centre and Lata Mangeshkar Hospital, Nagpur, IND

**Keywords:** glucose stability, plain vial, plasma glucose levels, preanalytical variable, serum blood glucose, sodium fluoride tubes

## Abstract

Objectives: Sodium fluoride (NaF) tubes are routinely recommended for blood glucose estimation because of their glycolysis-inhibiting properties. However, NaF inhibits enolase, a downstream glycolytic enzyme, and does not immediately prevent glucose consumption by blood cells. This raises the following questions: How does glucose stability compare between NaF plasma and plain serum over time? Does NaF offer better glucose preservation than plain serum? Does prompt separation of blood cells improve glucose stability? This study aimed to compare glucose stability in NaF plasma and plain serum at different time intervals and assess the effect of prompt cell separation on glucose preservation under routine laboratory conditions.

Materials and methods: This prospective observational study included 172 paired blood samples collected simultaneously in NaF and plain serum tubes. Samples were categorized into three groups: Group 1, plasma from NaF tubes; Group 2, serum from plain tubes; and Group 3, serum separated after centrifugation(using LabTop centrifuge at 3000 rpm for 10 minutes) and aliquoted into microcentrifuge tubes. Groups 1 and 2 were analyzed (using clinical chemistry analyzer Bonavera Chem 200, Biogeny Diagnostics Pvt. Ltd., Beacon Group, Surat, India) at <4 hours (baseline), four to six hours, 12-14 hours, and 24-26 hours after collection while stored at room temperature. Group 3 samples were analyzed after 24 hours. Percentage variation in glucose concentration from baseline was calculated.

Results: The mean difference between plain serum and NaF plasma within four hours of collection was 0.44%, with individual variation ranging from -0.62% to 3.36%. In NaF plasma, the mean decline in glucose concentration from baseline was 2.47% at four to six hours, 4.31% at 12-14 hours, and 9.97% at 24-26 hours. Corresponding individual variations ranged from 0.46% to 5.12%, 2.99% to 8.56%, and 3.96% to 17.47%, respectively. By contrast, the mean glucose decline in unseparated serum samples was 8.69%, 45.94%, and 75.59% at the respective time intervals. Glucose concentrations in aliquoted serum remained stable after 24 hours, showing <1% variation from baseline.

Conclusion: The consistent and substantial decline in glucose concentrations over time in both serum and plasma samples that remain in contact with blood cells highlights the critical importance of prompt cell separation to minimize pre-analytical glycolysis and ensure reliable glucose measurements. Notably, even when blood is collected in NaF tubes, delayed centrifugation and separation of plasma from blood cells may still permit considerable glucose loss. This raises concerns about the presumed advantage of fluoride-containing tubes over plain tubes when prompt sample processing is not ensured. In a hospital-based lab or referral lab with a rapid processing workflow, the use of a single plain serum tube can yield accurate glucose results, along with other biochemical tests, although it is not universally recommended as for peripheral collection centers. This can prevent the extra cost burden of fluoride vials for diagnostic labs and also decrease the extra burden of blood samples on patients. This also decreases the burden of biomedical waste generated in the form of fluoride vials, making it more sustainable and environmentally friendly. Further multicenter studies are warranted before recommending changes to existing specimen collection guidelines.

## Introduction

Accurate estimation of blood glucose is fundamental for the diagnosis and management of diabetes mellitus. Since laboratory glucose measurements form the basis of diagnostic criteria recommended by international organizations, minimizing preanalytical errors is essential to ensure reliable results [1]. Among the various preanalytical factors, continued glycolysis after blood collection remains one of the most important causes of falsely decreased glucose concentrations [2]. Blood cells, particularly erythrocytes and leukocytes, remain metabolically active after venipuncture and continue to consume glucose until serum or plasma is separated from the cellular components. Consequently, glucose concentration decreases progressively in unprocessed blood specimens, potentially leading to clinically significant errors in patient classification for diabetes, prediabetes, and non-diabetes and their management [3].

For more than five decades, blood collection tubes containing sodium fluoride (NaF), usually combined with potassium oxalate, have been recommended as the specimen of choice for glucose estimation because NaF inhibits glycolysis [4]. However, the inhibitory action of NaF occurs through inhibition of the enzyme enolase, which catalyzes a late step in the glycolytic pathway. Since earlier glycolytic reactions continue until intracellular intermediates accumulate, glycolysis is not immediately arrested after blood collection. As a result, glucose concentration may continue to decline during the first one to four hours despite the presence of NaF [4,5].

Several investigations have demonstrated that prompt centrifugation and early separation of serum or plasma from blood cells are more effective in preserving glucose concentration than the use of NaF alone [6]. Accordingly, recent laboratory guidelines have emphasized rapid specimen processing as an important measure to minimize glycolytic loss. Studies comparing serum obtained from plain tubes with plasma collected in NaF tubes have shown comparable glucose values when serum is separated without delay. These observations have challenged the long-standing belief that fluoride-containing tubes invariably provide superior glucose stability under routine laboratory conditions [7,8].

Despite this evidence, NaF tubes continue to be routinely used for glucose estimation in many diagnostic laboratories because of established laboratory practices and recommendations. In resource-limited settings, however, delays in specimen transport and processing are common, making it important to evaluate whether NaF tubes actually maintain glucose stability over time under routine laboratory conditions. Moreover, limited data are available from Indian laboratories comparing glucose stability in serum and fluoride plasma at different time intervals following sample collection.

Therefore, the present study was undertaken to compare serum glucose concentrations obtained from plain blood collection tubes with plasma glucose concentrations obtained from NaF tubes at different time intervals after sample collection. The study aimed to determine whether glucose concentrations remain stable in NaF tubes over time and whether serum obtained from plain tubes provides comparable results under routine laboratory practice.

## Materials and methods

This prospective observational study was conducted in the Department of Biochemistry at Uma Nath Singh Autonomous State Medical College, Uttar Pradesh, India. The study was conducted over a period of three months from July 2026 to September 2026. A total of 172 paired venous blood samples were collected for routine blood glucose estimation for this study. The blood glucose analysis was performed using blood samples that had already been collected from patients as part of their routine clinical management during hospital visits. The study used only those blood samples that were remaining after patients' routine tests. For each participant, venous blood was collected simultaneously into an NaF tube and a plain serum tube following standard phlebotomy procedures. Samples with visible hemolysis and inadequate volume were excluded. Blood samples in plain tubes were allowed to clot at room temperature. The blood samples collected in plain and fluoride vials were subsequently centrifuged to separate serum and plasma, respectively, from the cellular components. The separated serum and plasma were retained in their respective original vials at room temperature (23 °C to 26 °C) until testing was completed for all time intervals. Following centrifugation, a portion of the serum obtained from the plain collection tube was transferred to a sterile Eppendorf tube, while the remaining serum was retained in the original collection tube.

The collected samples were categorized into three groups: Group 1 (plasma obtained from NaF tubes), Group 2 (serum obtained from plain blood collection tubes), and Group 3 (serum in sterile Eppendorf tubes, which was separated and aliquoted immediately after centrifugation from plain tubes). 

All blood samples were maintained at room temperature (23 °C to 26 °C) throughout the study period. Samples of groups 1 and 2 were analyzed at four predefined time intervals, i.e., at <4 hours (baseline), four to six hours, 12-14 hours, and 24-26 hours after blood collection. Group 3 samples were analyzed after 24 hours of storage. Glucose concentration was estimated by the glucose oxidase-peroxidase (GOD-POD) method using an automated clinical chemistry analyzer (Bonavera Chem 200, Biogeny Diagnostics Pvt. Ltd., Beacon Group, Surat, India) with commercially available reagent kits provided by the same company.

Internal quality control was performed daily using two levels of quality control material, and calibration was carried out as per the recommended guideline. The laboratory performs internal quality control (IQC) using two control levels for glucose: Level 1 (normal) and Level 2 (abnormal). Over a period of two months, a total of 50 IQC observations were compiled for each control level to assess analytical imprecision and estimate precision-based measurement uncertainty. For IQC Level 1 (normal), the laboratory-derived mean glucose concentration was 115.09, with an SD of 4.84 and a CV of 4.21%. The QC provider-specified target value for Level 1 was 115, with an assigned SD of 8.5. For IQC Level 2 (abnormal), the laboratory-derived mean glucose concentration was 267.08, with an SD of 12.64 and a CV of 4.73%, while the QC provider-specified target value was 275, with an assigned SD of 20.5. The expanded measurement uncertainty was estimated from the within-laboratory imprecision, using the observed CV as the relative standard uncertainty and a coverage factor (k) of 2. The resulting expanded measurement uncertainty was 8.42% for IQC Level 1 and 9.46% for IQC Level 2. These values represent precision-based estimates of analytical measurement uncertainty derived from the available IQC data, while the QC provider's target values and assigned SDs provide additional reference points for assessing the laboratory's analytical performance. After processing the samples, glucose levels were measured in samples from all groups at predetermined time intervals. The glucose values for all groups at the specified time intervals were recorded in an Excel spreadsheet, and all statistical analyses were performed using Microsoft Excel (Microsoft Corp., WA, USA). The mean glucose concentration for each sample category was calculated at the predefined time intervals, along with the percentage variation in the mean glucose concentration relative to the baseline value. The percentage variation was calculated using the following formula:

*Percentage variation = Baseline mean glucose &minus; Mean glucose at time t variation / Baseline mean glucose &times; 100*

The primary outcome was the percentage variation in mean glucose concentration at each predefined time interval relative to the baseline mean glucose concentration. A significant variation in glucose concentration was observed even in plasma samples.

Approval from the Institutional Ethics Committee of Uma Nath Singh Autonomous State Medical College, Jaunpur, was obtained before the commencement of the study (IEC code-2026-015).

## Results

The mean glucose values for all samples at each time interval are presented in Table 1. A progressive time-dependent change in glucose concentration was observed in serum samples retained in the original plain vial. The mean glucose concentration in the serum samples decreased from 148.44 mg/dL at baseline to 135.68 mg/dL at four to six hours, 77.72 mg/dL at 12-14 hours, and 38.25 mg/dL at 24-26 hours. By contrast, glucose concentrations in plasma from the fluoride vial remained comparatively stable over the same observation period. The mean plasma glucose concentration was 147.81 mg/dL at baseline and 144.19, 141.32, and 133.07 mg/dL at four to six, 12-14, and 24-26 hours, respectively. The baseline sample was defined as the sample analyzed within <4 hours of blood collection. At baseline, the mean glucose concentration was 148.44 mg/dL in serum from the plain vial and 147.81 mg/dL in plasma from the fluoride vial, corresponding to a mean variation of only 0.44%, indicating less than 1% variation between the two sample types. 

**Table 1:** Mean glucose values for all samples at each time interval

Time interval	Mean value in plasma	Mean value in serum	Mean value in the aliquoted serum sample
Before four hours (baseline)	147.81 mg/dL	148.44 mg/dL	--
Four to six hours	144.19 mg/dL	135.68 mg/dL	--
12-14 hours	141.32 mg/dL	77.72 mg/dL	--
24-26 hours	133.07 mg/dL	38.25 mg/dL	148.81 mg/dL

The percentage variations in mean glucose concentrations in both sample types relative to the baseline are presented in Table 2. Compared with the baseline value, the mean glucose concentration in serum showed variations of 8.69%, 47.64%, and 75.59% at the different predefined time intervals, whereas the corresponding variations in plasma were 2.47%, 4.31%, and 9.97%, respectively. 

**Table 2:** Percent variations of the mean value from the initial value

Time interval	In plasma	In serum
Four to six hours	2.47%	8.69%
12-14 hours	4.31%	47.64%
24-26 hours	9.97%	75.59%

An additional analysis was performed using serum that was aliquoted after separation from the cellular components. The mean glucose concentration in the aliquoted serum sample after 24 hours was 148.81 mg/dL. This value showed less than 1% variation from the initial mean glucose concentrations measured in both the plain-vial serum and fluoride-vial plasma samples. Overall, the findings demonstrated a progressive decline in glucose concentration in serum retained in the original plain vial with increasing time after blood collection, whereas glucose concentrations in fluoride plasma showed comparatively limited variation over the same period. The glucose concentration in aliquoted serum remained close to the baseline concentration after 24 hours.

These findings underscore the importance of early centrifugation and separation of serum to minimize glycolytic loss and maintain analytical accuracy. Overall, the results indicate that prompt processing of plain serum provides glucose measurements comparable to those obtained from NaF plasma within the first four hours after collection, while separated serum offers superior long-term stability compared with both NaF plasma and unseparated serum.

## Discussion

The present study evaluated the stability of glucose concentrations in NaF plasma, unseparated serum, and promptly separated serum stored at room temperature under routine laboratory conditions. The findings demonstrate a clear effect of delayed sample processing on glucose stability. Although NaF plasma provided substantially better preservation of glucose concentrations than unseparated serum, a progressive decline in glucose was observed with increasing storage time. By contrast, serum that was promptly separated from cellular components and stored in aliquots remained remarkably stable for up to 24 hours, with less than 1% variation from the baseline concentration. These findings emphasize that appropriate pre-analytical handling, particularly timely separation of blood cells, is critical for obtaining reliable glucose measurements [9,10].

The mean difference of only 0.44% in glucose levels between plain serum and NaF plasma tested within four hours shows that both sample types give very similar results when processing is not significantly delayed. This observation suggests that, under appropriate pre-analytical conditions, either serum or NaF plasma can provide comparable glucose results. Previous studies have demonstrated that differences between serum and plasma glucose are relatively small when specimens are appropriately collected and processed. However, the choice of specimen type alone does not eliminate the potential for glucose loss, as continued cellular metabolism after blood collection remains an important source of pre-analytical variation [3,11].

In the present study, glucose concentrations in NaF plasma decreased progressively with increasing delay in analysis, with mean reductions of 2.47% at four to six hours, 4.31% at 12-14 hours, and approximately 10% after 24 hours. This finding is consistent with the recognized limitations of NaF as an antiglycolytic agent. Although NaF is widely used for glucose preservation, its inhibitory effect on glycolysis is neither immediate nor complete. NaF primarily inhibits enolase, an enzyme involved in the later stages of the glycolytic pathway. Consequently, several glycolytic reactions can continue after blood collection before sufficient inhibition of glycolysis occurs.

An important mechanism is that after blood collection, red blood cells (RBCs) continue to take up and use glucose. Human RBCs contain glucose transporter 1 (GLUT1), which allows glucose to enter the cells [12]. NaF does not block this transporter and therefore cannot prevent glucose from entering RBCs. As a result, glucose can continue to be used by RBCs even in the presence of NaF, leading to a gradual decrease in glucose levels during delayed analysis. RBCs may remain viable in the collection tube for up to about 24 hours, allowing cellular activity to continue during storage. This explains why glucose levels may decrease in NaF plasma when analysis is delayed. Therefore, the presence of NaF in a collection tube does not mean that glucose breakdown stops immediately after blood collection [4,13].

The delayed inhibitory action of fluoride has been demonstrated experimentally. Chan et al. reported that fluoride-containing specimens continued to undergo glucose loss during the early period following blood collection, indicating that fluoride does not immediately suppress glycolysis [14]. Mikesh and Bruns subsequently demonstrated that the inhibitory effect of fluoride is delayed because enolase represents a relatively distal step in the glycolytic pathway [5]. These observations support the findings of the present study and indicate that NaF alone may be inadequate for maintaining glucose concentrations when blood specimens remain in contact with cells for prolonged periods at room temperature.

The marked reduction in glucose concentration observed in unseparated serum further demonstrates the importance of ongoing cellular metabolism. In the present study, glucose decreased by 8.69%, 45.94%, and 75.59% at four to six, 12-14, and 24-26 hours, respectively. The substantially greater reduction compared with NaF plasma indicates extensive glucose consumption when serum remains in prolonged contact with metabolically active blood cells. Erythrocytes and leukocytes continue to metabolize glucose after collection, and this cellular glucose utilization can result in substantial analytical bias when specimens are not promptly processed [6]. The magnitude of glucose loss observed in the present study is therefore clinically relevant because a pseudo-low glucose concentration may lead to underestimation of the patient's actual glycemic status and, in certain circumstances, contribute to misclassification of glucose tolerance or failure to identify diabetes mellitus [15,16].

The excellent stability observed in promptly separated serum represents an important finding of the present study. Serum stored in aliquots following early separation demonstrated less than 1% variation from baseline even after 24 hours. Separation of serum from the cellular components substantially reduces ongoing cellular glucose consumption and consequently minimizes glycolytic loss. Current laboratory recommendations emphasize the importance of prompt separation of blood cells from serum or plasma when accurate glucose measurement is required [1,10]. This finding is particularly relevant to laboratories that process large numbers of specimens or receive samples from peripheral collection centers, where immediate analysis may not be feasible. Prompt centrifugation followed by separation of serum into secondary aliquots may therefore provide more reliable preservation of glucose concentration than prolonged storage of unseparated blood, even when NaF is used.

The present findings are consistent with previous investigations demonstrating that conventional NaF-containing tubes do not provide immediate protection against glycolysis. Gambino et al. demonstrated that acidification of blood using citrate was more effective than NaF alone in preventing glycolytic glucose loss [2]. The greater effectiveness of citrate-containing systems is attributed to more rapid inhibition of glycolysis compared with fluoride alone. Subsequent studies have similarly demonstrated improved glucose preservation with citrate-based glycolysis inhibitors compared with conventional NaF tubes [17]. These findings suggest that, when immediate separation is not possible, collection systems incorporating more rapidly acting glycolysis inhibitors may provide superior preservation of glucose concentrations.

From a laboratory management perspective, the findings emphasize the importance of the pre-analytical phase in maintaining the accuracy of glucose estimation. Delayed processing is particularly problematic in laboratories receiving specimens from geographically distant collection centers or in settings where centrifugation and analysis cannot be performed immediately after collection. Current laboratory recommendations therefore emphasize either rapid separation of plasma or serum from cellular components or the use of collection systems capable of providing more immediate inhibition of glycolysis. Establishing standardized protocols for prompt centrifugation and specimen separation may substantially reduce pre-analytical glucose bias and improve the reliability of laboratory results.

The findings of the present study also have implications for the interpretation of apparently low glucose concentrations in delayed specimens. A reduced glucose concentration in a specimen that has remained unprocessed for several hours may not necessarily reflect the patient's true in vivo glucose concentration. Instead, the observed reduction may partly represent continued cellular glucose consumption after blood collection. This distinction is particularly important when glucose results are close to clinical decision limits, because even relatively modest pre-analytical losses may influence clinical interpretation.

Overall, the present study demonstrates that the timing of cellular separation is a major determinant of glucose stability during pre-analytical handling. Although NaF plasma provides better preservation of glucose than unseparated serum, it does not completely prevent glucose loss during prolonged storage at room temperature. This can be explained, at least in part, by the fact that NaF does not inhibit GLUT1-mediated glucose transport into erythrocytes and acts primarily by inhibiting enolase at a relatively late stage of glycolysis [2,13]. Consequently, erythrocytes can continue to take up and metabolize glucose after blood collection before glycolysis is sufficiently inhibited. In contrast, promptly separated serum demonstrated minimal variation in glucose concentration over 24 hours in the present study. Therefore, prompt centrifugation and separation of serum or plasma from cellular components should remain a fundamental component of pre-analytical glucose management, particularly when immediate analysis is not possible.

## Conclusions

The findings demonstrate that glucose concentrations decrease progressively in unseparated blood samples, even in NaF tubes, indicating that NaF does not immediately inhibit glycolysis. Although NaF plasma showed a slower decline than unseparated serum, glucose loss increased with storage time. By contrast, promptly separated serum or plasma maintained glucose stability. The comparable glucose concentrations observed in plain serum and NaF plasma during the first four hours suggest that plain serum can provide reliable results when processed promptly. Furthermore, the stability of separated serum supports its suitability for delayed analysis. Using a single plain serum tube for glucose estimation and other biochemical investigations may simplify sample collection, reduce phlebotomy-related discomfort, lower costs, and minimize biomedical waste, provided that timely centrifugation and separation are ensured.

However, this study was conducted under controlled laboratory conditions, which may limit the generalizability of its findings. Further multicenter studies involving larger and more diverse populations, different analytical platforms, and varied laboratory workflows are needed to validate these observations. Such evidence is essential before modifying existing recommendations for specimen selection in routine glucose estimation.
